# Plasmonic Magnesium Nanoparticles Are Efficient Nanoheaters

**DOI:** 10.1021/acs.nanolett.3c03219

**Published:** 2023-11-27

**Authors:** Claire A. West, Vladimir Lomonosov, Zeki Semih Pehlivan, Emilie Ringe

**Affiliations:** † Department of Earth Sciences, University of Cambridge, Downing Street, Cambridge CB2 3EQ, United Kingdom; ‡ Department of Materials Science and Metallurgy, University of Cambridge, 27 Charles Babbage Road, Cambridge CB3 0FS, United Kingdom

**Keywords:** magnesium, plasmonics, photothermal therapy, photothermal transduction

## Abstract

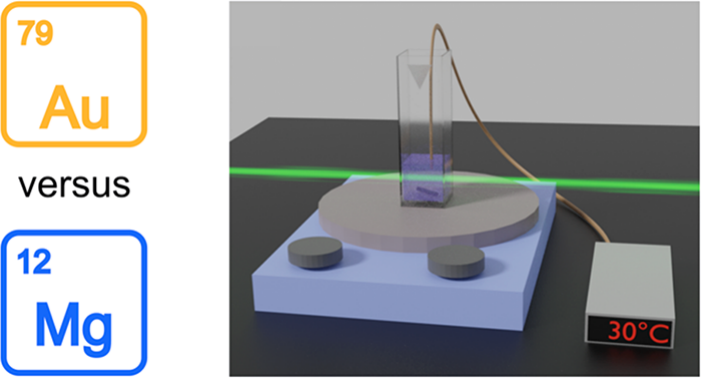

Understanding and guiding light at the nanoscale can significantly impact society, for instance, by facilitating the development of efficient, sustainable, and/or cost-effective technologies. One emergent branch of nanotechnology exploits the conversion of light into heat, where heat is subsequently harnessed for various applications including therapeutics, heat-driven chemistries, and solar heating. Gold nanoparticles are overwhelmingly the most common material for plasmon-assisted photothermal applications; yet magnesium nanoparticles present a compelling alternative due to their low cost and superior biocompatibility. Herein, we measured the heat generated and quantified the photothermal efficiency of the gold and magnesium nanoparticle suspensions. Photothermal transduction experiments and optical and thermal simulations of different sizes and shapes of gold and magnesium nanoparticles showed that magnesium is more efficient at converting light into heat compared to gold at near-infrared wavelengths, thus demonstrating that magnesium nanoparticles are a promising new class of inexpensive, biodegradable photothermal platforms.

A recent intersection of nanoscience and biology has fueled the emergence of a subfield wherein light-driven nanosources of heat are harnessed for temperature-mediated therapies. These so-called photothermal therapies have been envisioned to address a broad spectrum of ailments, including cancers,^1−3^ bacterial infections,^4,5^ atherosclerosis,^6^ retinal degradation,^7,8^ and cosmetic issues.^9,10^ The most well-studied of these treatments is photothermal cancer therapy,^11−22^ where nanoparticles (NPs) are delivered to a tumor site through active^23^ or passive^24^ targeting, the tumor is illuminated with light, the NPs transduce light into heat, and the tumor cells die due to hyperthermia.

Integral components to photothermal therapy are the NP’s high absorption cross section, low short-term impact, and minimal long-term toxicity. Materials that can meet the first two criteria include NPs of metals, semiconductors, carbon allotropes, rare earth doped oxides, and organic compounds.^25^ However, quantifying the long-term toxicity of these materials when injected intravenously remains challenging because while most NPs are processed by the kidneys and liver,^26^ some may remain in the body indefinitely. Biodegradable NPs can thus mitigate long-term toxicity concerns.

Mg is a biodegradable metal^27,28^ which can be synthesized into various NP shapes and sizes^29,30^ (stable in water when coated^31^) that sustain surface plasmon resonances.^32,33^ Plasmon resonances are tunable excitations of the free electrons in a material, which through nonradiative decay can produce heat.^34^ Au dominates plasmonic photothermal therapies (PPTT)^1^ because Au NPs support tunable plasmon resonances, their surface can be easily functionalized with, e.g., proteins, DNA, and enzymes^35,36^ for active targeting, drug delivery, and photodynamic therapies, and Au is one of the most chemically and physically inert metals. Multiple animal and human trials have validated the efficacy and short-term toxicity of Au NPs for PPTT.^37−40^ Mg-based PPTT is in its infancy. Several publications confirmed that Mg NPs are biodegradable,^41−44^ and animal experiments suggested its feasibility for use in PPTT.^44,45^ However, these investigations did not take advantage of Mg’s plasmonic character: NPs were illuminated far off-resonance, without analysis of shape effects and without proof that Mg remained metallic under the biological conditions.

Here, we aim to establish whether NPs of Mg, a newcomer in the plasmonics toolbox,^32,46−48^ can produce enough heat at low NP concentrations and laser intensities to be a biodegradable alternative for PPTT. We present numerical results comparing the photothermal properties of identically shaped Mg and Au NPs of various sizes. Then, we explore crystallographically correct shapes for Mg, followed by experimental measurements on both Au and Mg NP of different shapes and sizes illuminated at two different wavelengths. Our findings indicate that Mg NPs outperform Au in photothermal transduction at near-infrared wavelengths and is thus a competitive, biodegradable material for PPTT.

Photothermal properties, underpinning the viability of Mg for PPTT, were assessed by comparing the macroscopic properties of the Mg and Au. The two metals have different complex permittivities ϵ, a bulk parameter that describes the plasmonic capabilities of a material, due to their dissimilar electronic structures. Across visible through infrared wavelengths, the real part of ε, Re(ε), of Mg is more than twice that of Au (Figure 1A), i.e., Mg is more polarizable. Mg’s imaginary part, Im(ε), is an order of magnitude larger than that of Au for wavelengths greater than 530 nm (Figure 1B), indicating that Mg absorbs more of the incident electric field compared to Au.

**Figure 1:**
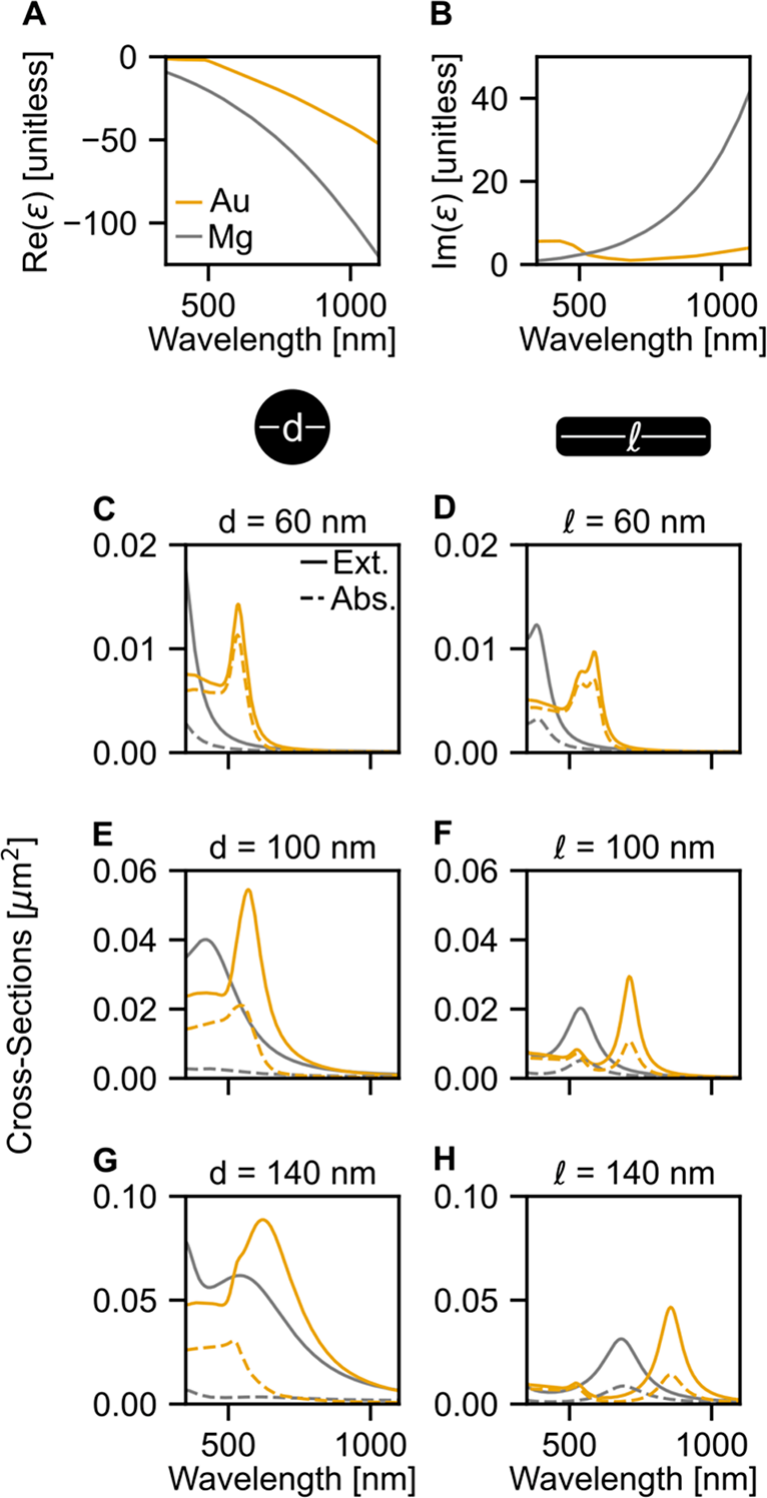
Impact of the differences in permittivity between Mg and Au. (A) Real and (B) imaginary complex permittivity of Au (gold trace) and Mg (gray trace). Extinction (solid) and absorption (dashed) cross sections of Mg and Au (C, D, G) nanospheres and (D, F, H) nanorods of indicated diameter (*d*) and length (). The rods are 40 nm in width.

The impact of differences in permittivity on the optical response was assessed by simulating the extinction and absorption cross sections of Mg and Au nanospheres (Figures 1C,E,G) and nanorods of fixed width of 40 nm (Figures 1D,F,H) in a uniform background of water using the discrete dipole approximation,^49^ with results averaged over six laser orientations (Figure S1). Note that while these identical shapes are not achievable experimentally due to the different crystal packings of Mg and Au, the purpose of these simulations was to isolate the impact of the permittivity on the optical response. For both Mg and Au NPs, as the NP size increases, the extinction cross section increases. However, for all NP shapes and sizes, the extinction cross sections of Mg NPs were lower compared to those of Au NPs of the same dimensions, and their resonances were blue-shifted and broadened. This indicates that Mg suffers from more damping than Au. The absorption cross sections follow the same trends as the extinction cross sections, except for significant differences in line shape between 350 and 600 nm because of Au’s interband transitions.

The ratio of absorption to extinction in plasmonic NPs is size-dependent, and NPs transition from primarily absorbing to primarily scattering as the size increases. This ratio is also modulated by the wavelength- and material-dependent Im(ε). Apart from the interband region of Au (less than 530 nm) where absorption dominates, the absorption to extinction ratios in Au and Mg decrease for increasing NP size and increase as wavelength increases (Figure S1).

After light is absorbed by a NP and a plasmon is excited, the electrons quickly dephase and couple to phonon modes, raising the temperature of the NP and subsequently heating the local environment.^34^ For a single nanosphere in a uniform medium, the steady-state temperature rise Δ*T* resulting from the light absorption is
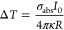
1where σ_abs_ is the absorption cross-section, *I*_0_ is the intensity of the laser, κ is the thermal conductivity of the environment, and *R* is the radius of the nanosphere. Thus, two nanospheres made of different materials will produce the same amount of heat power if they have the same absorption cross section. Further, if the nanospheres are identical in size and absorption cross section, they will achieve the same steady-state temperature. This means that the steady-state temperature of the NP is proportional to the absorption cross section simulated in Figures 1C–H, and so the relative magnitude and line shape of the dashed traces dictate the temperature difference between the Au and Mg.

In addition to differences in their bulk properties, Au and Mg also form different crystal structures. Au (and most other plasmonic metals) crystallizes in face-centered cubic, and Mg crystallizes in hexagonal close-packed. This lattice packing dictates the crystal geometries obtainable in the NP syntheses. Each geometry will not only have different optical and thermal properties but also have different cell–particle interactions (smaller, spherical NPs have shown better cellular uptake^50,51^) that may impact PPTT efficacy.

Synthesized Mg NPs can be grouped into two classes of shapes: faceted spheroids^29^ and hexagonal platelets,^30,52^ the latter comprising both single crystalline and elongated twinned platelets. These shapes, emerging from different synthetic approaches, possess different size-dependent optical and thermal properties. To investigate these effects, we modeled faceted spheroids, single-crystalline hexagonal platelets, and elongated twinned hexagonal platelets (Figure 2) with 50, 100, 150, and 200 nm lengths, each with a 10 nm MgO layer consistent with experimental observations.^32,52,53^ The absorption cross sections of the three shapes with four different sizes were calculated with the NPs in an infinite background of isopropyl alcohol (Figure 2A–C). The optical results were averaged over six laser orientations to match experimental conditions. Each NP supports plasmon resonances in the visible through near-infrared wavelengths. The resonances red-shift with increasing size as retardation begins to take effect. The faceted spheroid supports damped plasmon modes, while the higher eccentricity shapes have sharper resonances, as expected.^54^

**Figure 2:**
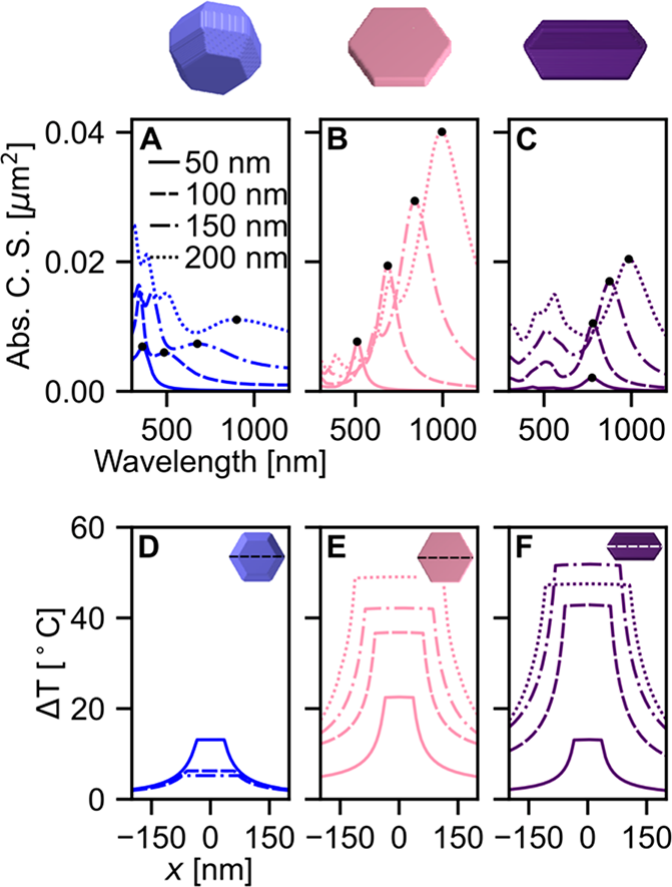
Effects of NP shape on the plasmonic response of faceted spheroids (blue), single crystal hexagonal platelets (pink), and elongated twinned platelets (purple). (top) Model of the NP shapes. (A–C) Absorption cross sections (C. S.) of each NP shape, calculated for four NP diameters: 50 nm (solid), 100 nm (dashed), 150 nm (dash dot), and 200 nm (dotted), where diameter is defined as the longest tip-to-tip distance indicated by the dashed line in panels D–F. (D–F) Steady-state temperature line sections through the center of each NP calculated with excitation at the dipole resonance in a background of isopropanol.

The temperature of an arbitrarily shaped NP in a uniform background may be approximated using eq 1, where *R = R*_eff_ and *R*_eff_ is the effective radius of a sphere of the same volume,^55^ making the temperature increase depend on both the absorption cross section and volume of the NP. For more precise temperature calculations, the thermal discrete dipole approximation^56^ framework was used here (Figure S1). The temperature of each NP was calculated at the dipole resonance under longitudinal laser polarization (Figures 2D–F). The results show that for smaller NPs (50 nm), the hexagonal platelet (pink) reaches the highest temperature when driven at the dipole resonance, and the elongated twinned platelet (purple) and hexagonal spheroid (blue) reach comparable temperatures. As NP size increases, the faceted spheroid temperature decreases, the temperature of the hexagonal platelet increases, and that of the twinned elongated platelet first increases and then decreases. These trends result from the absorption cross sections increasing and the scaling of the volume increasing at different rates depending on the NP shape. For the largest NP (200 nm), the twinned elongated platelet and the hexagonal platelet reach similar temperatures, much larger than that of the 200 nm faceted spheroid.

The temperature of an NP suspension can be modeled as the sum of the temperature contributions from all NPs illuminated in the suspension. Note that when modeling the electromagnetic response of a suspension, it is often appropriate to only consider the optical properties from a single NP. However, due to the much larger length scale of thermal diffusion, all heat-producing NPs in the suspension must be considered. The temperature of nanosphere *i* suspended in a solution with *N* other nanospheres is^57^. Thus, the single particle data presented above can be used to compare the behavior or different colloids, as the temperature of an NP suspension of identical Mg (or Au) NPs will reach a temperature that is primarily dependent on the power each NP absorbs.

However, for a real NP suspension, this approach is in some cases too simplistic. Suspensions of different NPs may aggregate and/or sediment at different rates; both effects affect the maximum temperature. Also, size and shape heterogeneity adds to the complexity of the calculation. In such cases, experimental measurements allowing the calculation of the photothermal transduction efficiency are a valuable comparison platform.

The photothermal transduction efficiency, η, is determined by measuring the temperature of an NP suspension undergoing laser-induced heating followed by (laser-free) cooling. The temperature change, , of the NP suspension is related to the heat power generated by the NPs, *P*_np_, generated by the system (cuvette and solvent), *P*_sys_, and leaving the cuvette, *P*_ext_, through conservation of energy:^58−60^
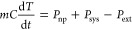
2where *m* and *C* are the mass and specific heat capacity of the NP suspension (which can be approximated as the *m* and *C* of the solvent given the mass disparity). *P*_np_ is determined as the difference between incident power *P*_0_ and transmitted power *P*_trans_ converted into an absorbed power by multiplying by η. Other terms are defined, and experimental details are provided in Figure S3.

Suspensions of different NP material, shape, and size have different wavelength-dependent η. Larger values of η indicate that the NPs are more efficient at converting light into heat and therefore potentially better for PPTT. To compare the performance of Mg that of Au, we determined η for various experimental Mg and Au samples. Small faceted Mg spheroids were synthesized using a one-step reduction of a Mg precursor (di-*n*-butylmagnesium) with Li_2_Biphenyl. The larger faceted spheroids and Mg hexagonal platelets were synthesized via a seed-mediated growth approach (Figure S3).^29^ Sizes range from 40 to 200 nm in diameter with a self-limiting 10 nm surface oxide layer. The thickness and self-limiting nature of this oxide on equivalent NPs was previously characterized using scanning transmission electron microscopy (STEM), electron energy loss spectroscopy,^32,52^ STEM energy-dispersive X-ray spectroscopy,^32,33,52^ and X-ray diffraction.^53^

We used citrate-coated Au nanospheres in water (Figures 3A–C and Figure S3) with diameters of 11, 48 (both synthesized in-house), and 150 nm (purchased from Sigma-Aldrich) and polyvinylpyrrolidone (PVP)-coated Mg NPs in isopropanol, specifically faceted spheroids of 38, 150, and 202 nm in diameter, and hexagonal platelets of 200 nm diameter with a 2:1 ratio of single crystal to elongated twinned hexagonal platelets (Figures 3D–G and Figure S2). The size distribution, average length, and standard deviation of each suspension are listed in Figure 3. The concentration was varied from 30–60 μg/mL for the Au and 10–40 μg/mL for the Mg suspensions, with absorbance values between 0.5–2.5 at 532 nm (normalized in last row of Figure 3 and not normalized in Figure S3). The extinction of the Au NP suspensions indicates a resonance at 520–540 nm that red-shifts and broadens for increasing NP size. The extinction of the Mg NP suspensions was broader due to the larger distribution of size and shape in each sample, aggregation, and uneven surface oxidation. As the Mg NP suspensions increase in size, multiple peaks emerge, as predicted by the higher order modes obtained numerically (Figure 2).

**Figure 3:**
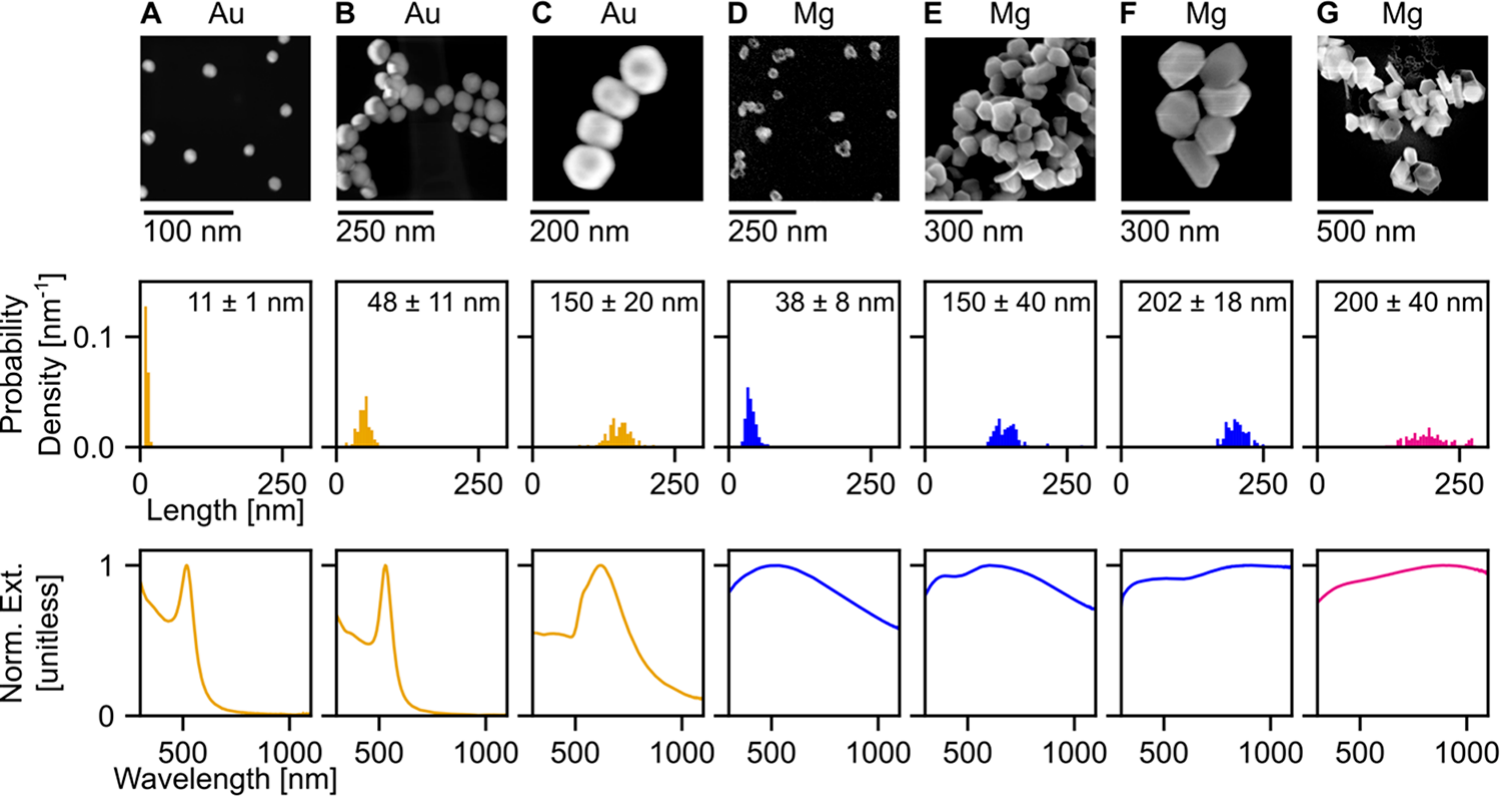
Optical characterization of (A–C) Au and (D–G) Mg NP suspensions. The first row contains (A, B) representative scanning transmission electron microscopy (STEM) and (C–G) scanning electron microscopy (SEM) images. The second row shows the length distribution of NPs in each suspension with the averages and standard deviations indicated. The last row presents the experimentally measured normalized extinction of each NP suspension.

η was extracted by measuring the temperature change of the NP suspensions, under stirring, induced by laser illumination and equilibration with the environment (Figure S3). The resulting efficiencies depend on the size, shape, and NPs material as well as on the wavelength of laser illumination (Figure 4). The size and shape dependence of η is as expected: as NP size increases, NPs scatter more than absorb, and as NP shape increases in eccentricity, NPs absorb more than scatter (Figure 1). Such changes in the ratio of light absorption to extinction directly impact the photothermal efficiency achievable by a NP suspension; i.e., as NP size increases, η decreases, and as shape increases in eccentricity, η increases.

**Figure 4:**
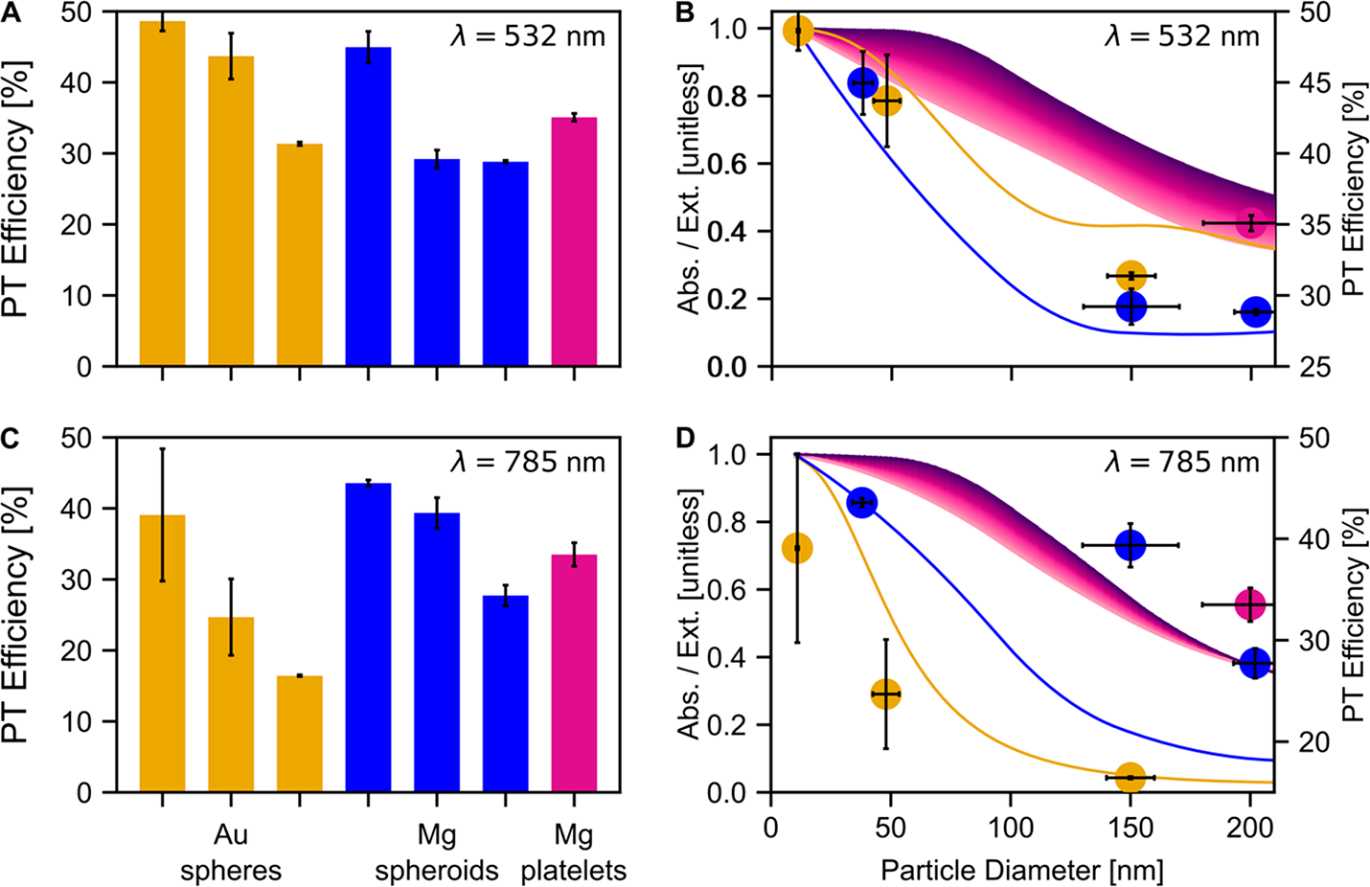
Photothermal (PT) efficiency measurements for the Mg and Au NP suspensions. Photothermal efficiency of each NP suspension (left to right: 11, 48, and 150 nm Au spheres, 38, 150, and 202 nm Mg spheroids, and 200 nm Mg platelets) at (A) 532 nm and (C) 785 nm. The height of each bar corresponds to the average photothermal efficiency, and the error bars correspond to the standard deviation, evaluated across multiple measurements. Efficiencies replot (dots) with simulation of absorption/extinction overlaid (line traces) at (B) 532 nm and (D) 785 nm. The *x*-axis error bars on the experimental data represent the standard deviation of the size distribution of the NP suspensions.

The wavelength-dependent differences in η between Mg and Au NPs are evident when comparing η obtained upon 532 and 785 nm laser excitation (Figure 4). At 532 nm, the Au nanospheres were more efficient than the Mg faceted spheroids: η_Au Sph_ = 49, 44, and 31% for 11, 48, and 150 nm diameter, respectively, compared to η_Mg Sph_ = 45, 29, and 29% for 38, 150, and 202 nm diameter, respectively. The Mg hexagonal platelets were more efficient than the largest Au nanosphere and the two larger Mg faceted spheroids (η_Mg Plate_ = 35%). However, at 785 nm, Mg NPs were on average more efficient than those of Au for both Mg NP shape categories (η_Au Sph_ = 39, 30, and 16% compared to η_Mg Sph_ = 44, 39, and 28% and η_Mg Plate_ = 34%). These trends are not due to the extinction of Au NPs being largest at 500–600 nm and nearly zero at 800 nm, while the Mg NPs have a broad extinction profile across 500–800 nm (Figure 3). Instead, they are a consequence of differences in the permittivity between the two materials as well as the quality of the plasmon mode.

The photothermal efficiencies (Figures 4A,C, replotted as dots in Figures 4B,D) agree well with the ratio of simulated absorption to extinction cross sections (lines in Figures 4B,D). The simulations show Au nanospheres, Mg spheroids from the first column of Figure 2, and an interpolation between hexagonal platelets and elongated twinned platelets, second and third columns of Figure 2. The large standard deviation of the 11 nm Au nanospheres at 785 nm is a consequence of the low absorbance values (approximately 0.01 at 785 nm versus 0.6 at 532 nm), causing the efficiency to be more sensitive to temperatures variations. The difference between experimental photothermal efficiency and the numerical absorption/extinction of the large Mg spheroids is likely due to laser-induced aggregation, increasing the local concentration of NPs along the path of the laser and producing more heat than predicted.

Of the experimentally studied Mg NP suspensions, the 38 nm faceted spheroids had the largest η and generated sufficient temperature increases (5 °C) for PPTT.^40^ The photothermal stability of this suspension was measured under conditions identical with those in Figure 4, over the course of five heating and cooling cycles (Figure 5). After five cycles spanning over 3 h, the maximum temperature reached after 20 min of heating remained stable, indicating stability of the photothermal performance. Experimental results for other NP suspensions illuminated at 532 nm indicate a similar level of stability (Figure S4).

**Figure 5:**
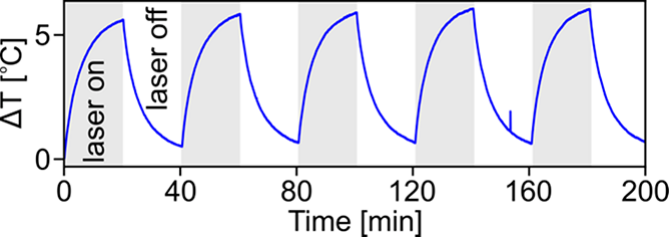
Stability of 38 nm diameter Mg faceted spheroids after five cycles of laser heating at 785 nm. Gray regions indicate laser on, and white regions indicate laser off.

In conclusion, Mg NPs support localized surface plasmon resonances that can be controlled by changing the NP size and shape, enabling the NPs to strongly absorb from the ultraviolet through the infrared wavelengths. When illuminated at wavelengths near their plasmon resonances, NPs produce appreciable heat. Herein, we simulated the photothermal properties of identical Au and Mg nanospheres and nanorods and crystallographically relevant shapes of Mg. We then compared the heat produced by Au and Mg NP suspensions and found that Mg faceted spheroids were more efficient at converting light to heat compared to Au nanospheres at infrared wavelengths. Furthermore, the Mg spheroids sustained this photothermal capacity for five successive rounds of laser-induced heating and cooling. The performance (Δ*T* > 5 °C at 3 W/cm^2^ with 785 nm illumination and 0.5 μM concentration) and stability of Mg NPs make them an alternative material which offers attractive biocompatibility for PPTT.

## Supporting Information Available

Computational details and supplementary optical simulations, additional images of Au and Mg NPs, details of the photothermal transduction model, raw data, and stability measurements (PDF)
