# Supplementary material for: Plasmonic Magnesium Nanoparticles Are Efficient Nanoheaters
Source: Nano Lett. 2023 Nov 27;23(23):10964–70. doi: 10.1021/acs.nanolett.3c03219 (PMC10722534; doi:10.1021/acs.nanolett.3c03219)
Supplement: Supplementary file 1 — nl3c03219_si_001.pdf [file nl3c03219_si_001.pdf]

**Supporting Information**

# Plasmonic Magnesium Nanoparticles are Efficient Nanoheaters

Claire A. West[1], Vladimir Lomonosov[1], Zeki Semih Pehlivan[1], Emilie Ringe[1*]

1. Department of Earth Sciences, University of Cambridge, Downing Street, Cambridge CB2 3EQ, United Kingdom and Department of Materials Science and Metallurgy, University of Cambridge, 27 Charles Babbage Road, Cambridge CB3 0FS, United Kingdom

*er407@cam.ac.uk

**Section S1: Computational Details and Additional Simulations of Au and Mg Nanospheres and Nanorods**

Optical calculations were performed using the discrete dipole approximation (DDSCAT 7.3),[1] an approach where Maxwell's equations are solved on a discretized shape of electromagnetically coupled point dipoles. In Figure 1, the shape of the Au and Mg NPs were modeled as spheres and rods. In Figure 4, the Au NPs were modeled as spheres, while the Mg NPs were modeled using a Wulff construction (Crystal Creator [2,3]) and images of the NPs. Steady-state temperature calculations were performed using the thermal discrete dipole approximation (*t*-DDA),[4] that solves the heat diffusion equation on the thermally (and electromagnetically) coupled points, sourced by the fields and polarizations output from DDSCAT. *t*-DDA outputs both the temperature of the target and the environment through Green's function projection operations (as described in Ref. 4). The permittivity of the materials used in the calculations were Palik[5] for Mg, Johnson and Cristy[6] for Au, a constant 1.7 for MgO, and a constant 1.3771 for isopropanol. For the thermal calculations, the intensity used was $I_0 = 10^8$ W/m$^2$, and the thermal conductivities were 0.137 W/mK for the background material (isopropanol), 156 W/mK for Mg, and 42 W/mK for MgO.

Direct comparison between the absorption and extinction cross-sections of Au and Mg nanospheres and nanorods indicates that the ratio of light absorption to extinction is size-, material-, and shape-dependent (Figure 1, Figure S2-1, and Figure S2-2). The nanosphere absorption is saturated by the interband transitions in Au. For nanorods, Au and Mg both support a sharp resonance which redshifts and increases in intensity with increasing nanorod length. At equivalent lengths, Au nanorods have sharper and higher in amplitude resonances. Yet, the ratio of absorption to extinction is larger for Mg compared to Au at wavelengths greater than 550 nm.

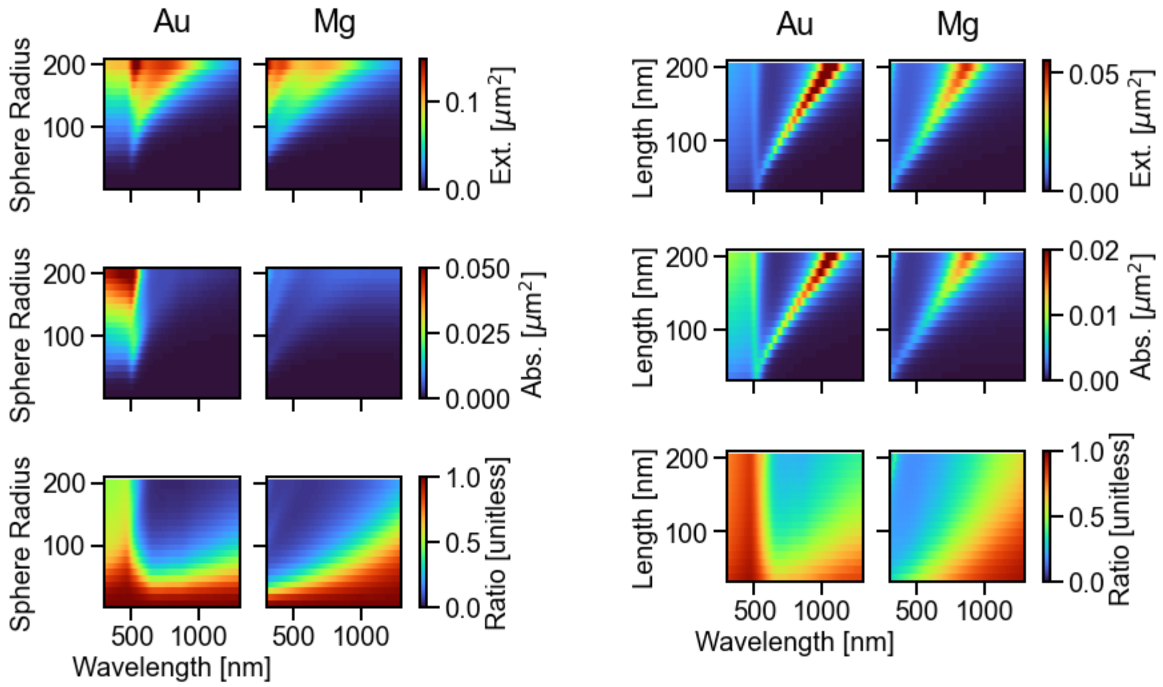


**Figure S1-1**. Simulated optical spectra of Au and Mg (A) spheres and (B) rods of diameters 50 nm – 200 nm. (Row 1) Extinction cross-section, (row 2) absorption cross-section, and (row 3) ratio of absorption to extinction cross-section of Au and Mg nanospheres and nanorods.

## Section S2: Characterization Details and Additional Images of Au and Mg NPs

The 11 nm and 48 nm Au NP samples were drop cast onto a Cu-supported lacey ultrathin carbon membrane for scanning transmission electron microscope (STEM) imaging, acquired at 200 kV on a FEI Osiris STEM (Figure 3A,B and Figure S2-1 A-D). The remaining Au and Mg NP samples were drop cast onto silicon wafers for scanning electron microscopy (SEM) imaging, performed on a FEI Nova NanoSEM, operated at 5 kV and equipped with an in-lens detector for secondary electron imaging. (Figure 3C-G, Figure S2-1 E,F, and Figure S2-2).

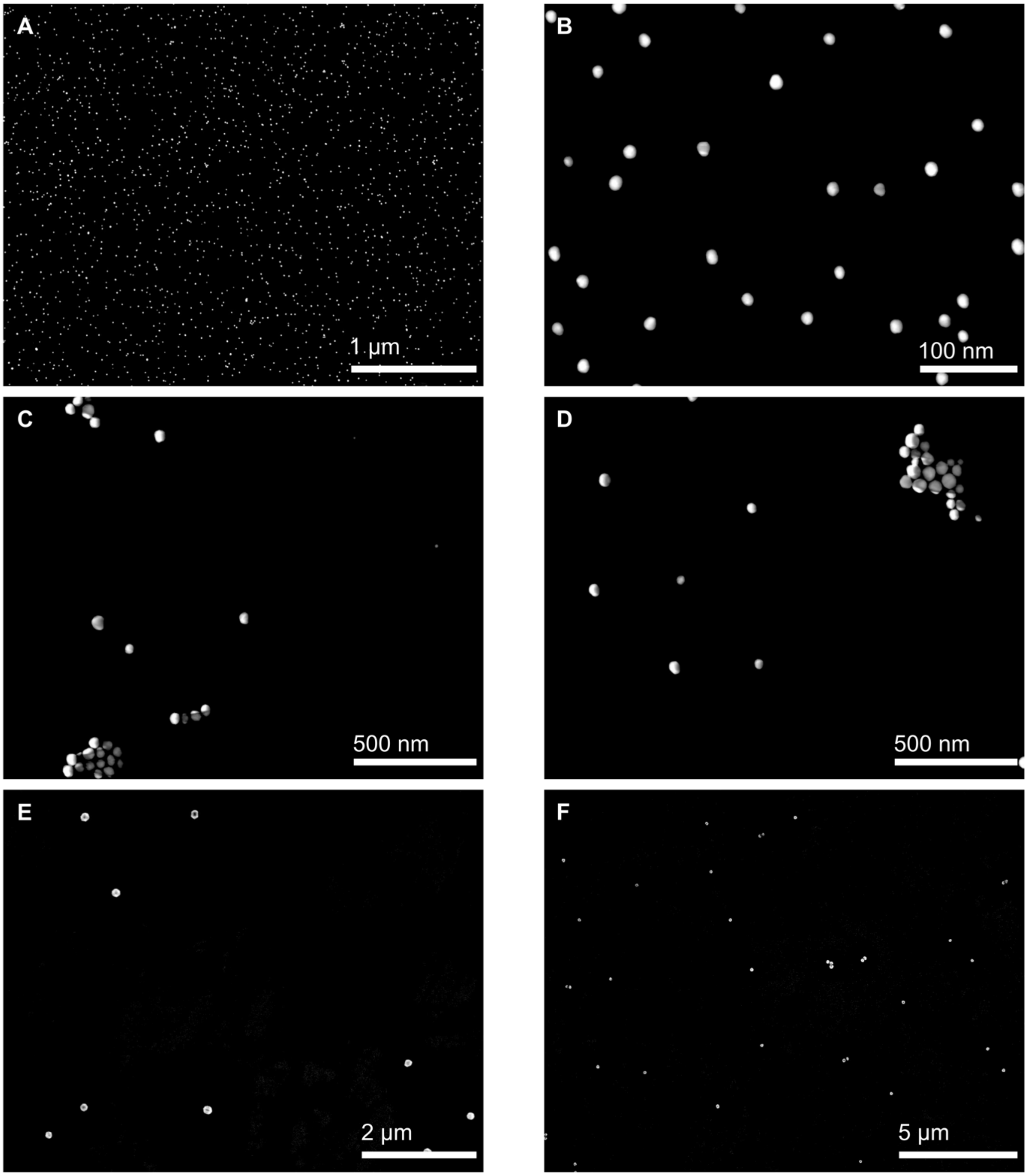


**Figure S2-1.** Images of Au nanospheres of average diameter of (A-B) 11 nm, (C-D) 48 nm, and (E-F) 150 nm.

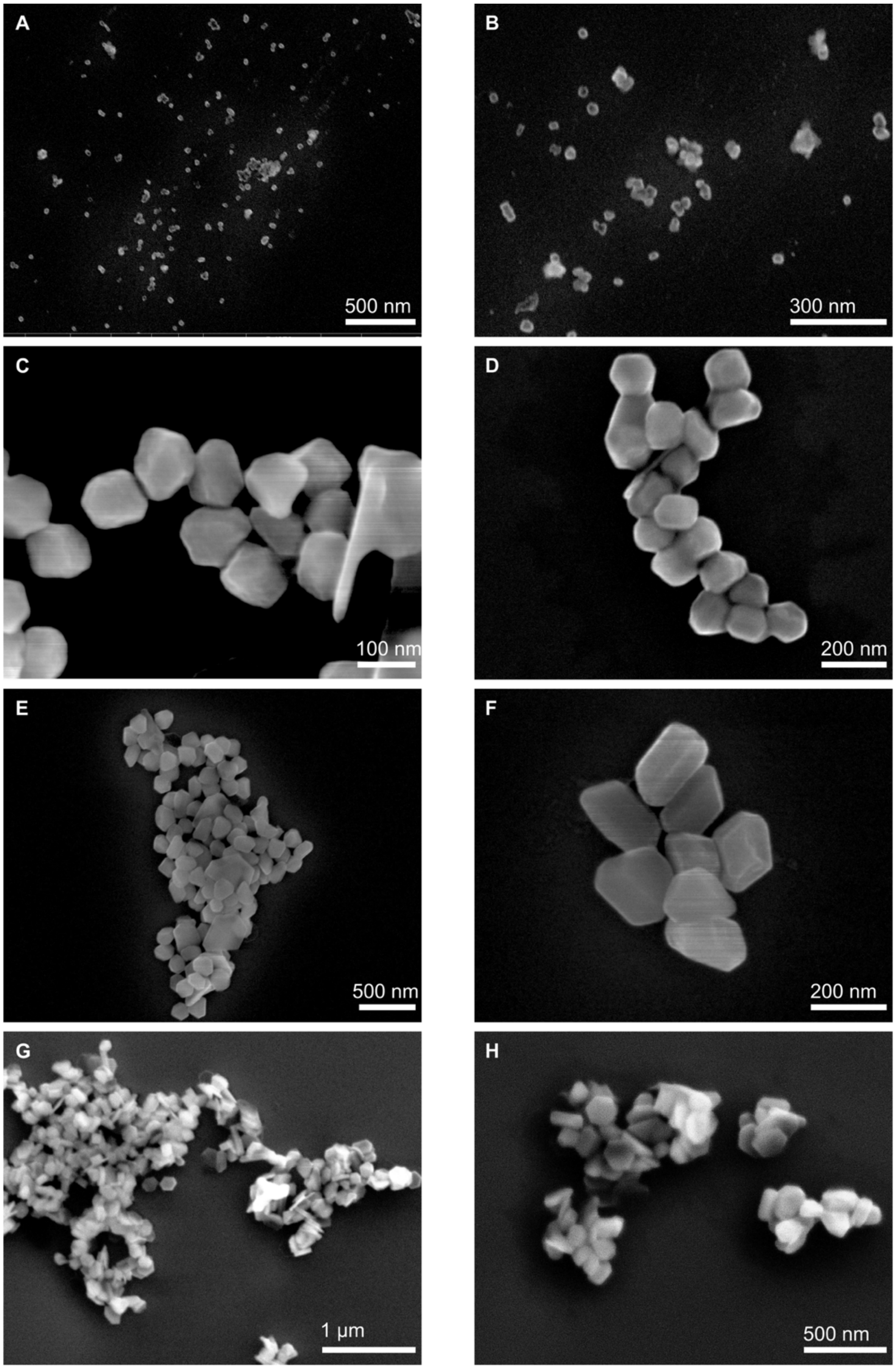


**Figure S2-2**. Images of Mg faceted spheroids of average diameter (A-B) 38 nm, (C-D) 150 nm, (E-F) and 202 nm, and Mg hexagonal platelets of average diameter (G-H) 200 nm.

**Section S3: Photothermal Transduction Model and Experimental Details**

$P_{\text{sys}}$ and $P_{\text{ext}}$ were determined by assuming the system follows Newton's law of cooling: $P_i = h_i S(T_i^{\text{max}} - T_i^0)$ where $i$ represents the total heat leaving the cuvette (ext) or the heat produced by all components of the system except the NPs (sys). $h_i$ is the heat transfer coefficient, $S$ is the surface area of the NP suspension in the cuvette, $T_i^{\text{max}}$ is the maximum steady-state temperature reached by the liquid when illuminated with a laser, and $T_i^0$ is the minimum temperature once the laser has been turned off and the system returns to steady-state.

Determining $\eta$ for the different NP suspensions is done in three steps: 1) measure the absorbance of the NP suspension at the wavelength which will be used for laser illumination to determine $P_{\text{np}}/\eta$, 2) measure the temperature of the solvent as it heats (laser on) and cools (laser off) to determine $h_{\text{sys}}$ and therefore $P_{\text{sys}}$, and 3) repeat the measurement with the NPs suspended in the solvent to determine $h_{\text{ext}}$ and $P_{\text{ext}}$. The heat transfer coefficients ($h_i$) are determined by fitting the measured temperature cooling curves to the solution of Eq (2) (separately for sys and ext, and $P_{\text{np}} = 0$):

$$T_i(t) = T_i^0 + (T_i^0 + T_i^{\text{max}})e^{-t\frac{h_i S}{mC}} \qquad \text{(S3-1)}$$

where $m$ is the mass of the solvent, and $C$ is the specific heat capacity (estimated to be the specific heat capacity of the solvent). Therefore, $\eta$ is

$$\eta = \frac{P_{\text{ext}} - P_{\text{sys}}}{P_0 - P_{\text{trans}}} = \frac{h_{\text{ext}} S(T_{\text{max}} - T_0) - P_{\text{sys}}}{P_0(1 - 10^{-A})}. \qquad \text{(S3-2)}$$

The raw data used to determine $P_{\text{sys}}$ is shown in Figure S3-1, at laser wavelength (A) 532 nm and (B) 785 nm for (blue) water and (orange) IPA. This is the minimal heat power produced when the laser illuminates the cuvette containing only the solvent. The temperature cooling data was fit to Eq. (S3-1), as indicated in the dashed black trace, and $P_{\text{sys}}$ was determined using the equation

above. The dashed lines indicate the average value, which was then used to calculate $\eta$. Note that across the photothermal transduction literature[7,8] the heat power terms in Eq. (S3-2) and Eq. (2) are sometimes referred to as heat fluxes or rates of heat transfer, yet given the units are energy per time, we will refer to these terms as heat powers.

Citrate-capped 11 nm Au NPs were prepared using a modified Turkevich synthesis.[9] The 11 nm Au NPs were then used to produce the 48 nm Au NPs using a seed-mediated growth method.[10] The 150 nm Au NPs were purchased from Sigma-Aldrich, Au NPs 150 nm diameter, stabilized in a suspension in citrate buffer.

The following materials were used in the synthesis of the Mg Ns: Li pellets (99%), naphthalene, biphenyl, 1.0 M di-n-butylmagnesium in heptane, 0.7M n-Butyl-sec-butylmagnesium poly(vinyl pyrrolidone) (PVP, MW 10,000), anhydrous tetrahydrofuran (THF) and anhydrous isopropanol (IPA), all purchased from Sigma-Aldrich and used as supplied. All glassware was washed with nitric acid and flame-dried under vacuum before use.

Mg NPs were synthesized using $Li_2$Napht or $Li_2$BiPhenyl dianions as reducing agents. A typical procedure for $Li_2$Napht formation was as follows: 0.028 g of Li pellets (4.05 mmol), 0.26 g of naphthalene (2.03 mmol), 20 mg PVP (0.18 mmol monomer) and 10.75 mL of degassed anhydrous THF were added to a 25 mL Schlenk flask under Ar atmosphere and sonicated for 45 minutes (Allendale Ultrasonics, 100 W 3 L). $Li_2$BiPhenyl was prepared following the same protocol using 0.32g of biphenyl instead of naphthalene. Small faceted spheroids were synthesized using a one-step reduction of a di-n-butylmagnesium with $Li_2$Biphenyl. In a typical synthesis of Mg NPs, 1.75 mL of di-n-butylmagnesium was injected into freshly prepared reducing agent under vigorous stirring and the reaction was allowed to proceed for 18 hours at room temperature (20°C) before quenching with 2 mL of anhydrous IPA. The larger faceted spheroids were synthesized

using seed-mediated growth approach. Mg NPs synthesis was initiated by injecting 1.75 mL of di-n-$MgBu_2$ in heptane (1.0 M) into freshly prepared $Li_2$Napht solution followed by addition of 2 mL of naphthalene in THF (1.0 M) after 5 minutes of reaction, to convert all unreacted $Li_2$Napht to LiNapht. The resulting mixture was left to react for 60 minutes and then quenched with 2 mL of IPA. The size of the larger faceted spheroids was increased by addition of a second identical dose of Mg precursor 30 minutes after the naphthalene injection and letting the mixture to react for a further 18h. The second class of NPs, hexagonal platelets, was synthesized using a similar seed-mediated growth approach, but with n-butyl-sec-butylmagnesium as the Mg precursor and reaction was quenched 60 min after naphthalene injection. In all synthesizes the solid product was recovered by centrifugation and residual by-products were removed by centrifugation and redispersion steps in anhydrous IPA twice, anhydrous THF twice and anhydrous IPA twice, before redispersing in anhydrous IPA.

The photothermal transduction experiments (results in Figures 4-5 and Figures S3-1-3) were performed with 1 mL of a NP suspension (Au NPs in water or Mg NPs in anhydrous isopropanol) in a standard polymethyl methacrylate (PMMA) cuvette, under stirring. A K-type thermocouple was inserted into the cuvette, and the temperature was recorded. A 532 nm laser (Cobolt Samba, HÜBNER Photonics) and a 785 nm laser (Innovative Photonic Solutions), both set to a power of $100 \pm 5$ mW and a beam diameter of 2 mm, illuminated the NP suspension within the cuvette. The NP suspension was illuminated for 20 minutes, then the laser was then turned off and the temperature was recorded for another 20 minutes as it cooled. Additional photothermal stability measurements (Figure S4-1) were performed with 0.5 mL of NP suspension in a micro-PMMA cuvette, not under stirring, with the identical thermocouple and laser. Absorbance measurements were performed using the Evolution 200 Series Spectrophotometer.

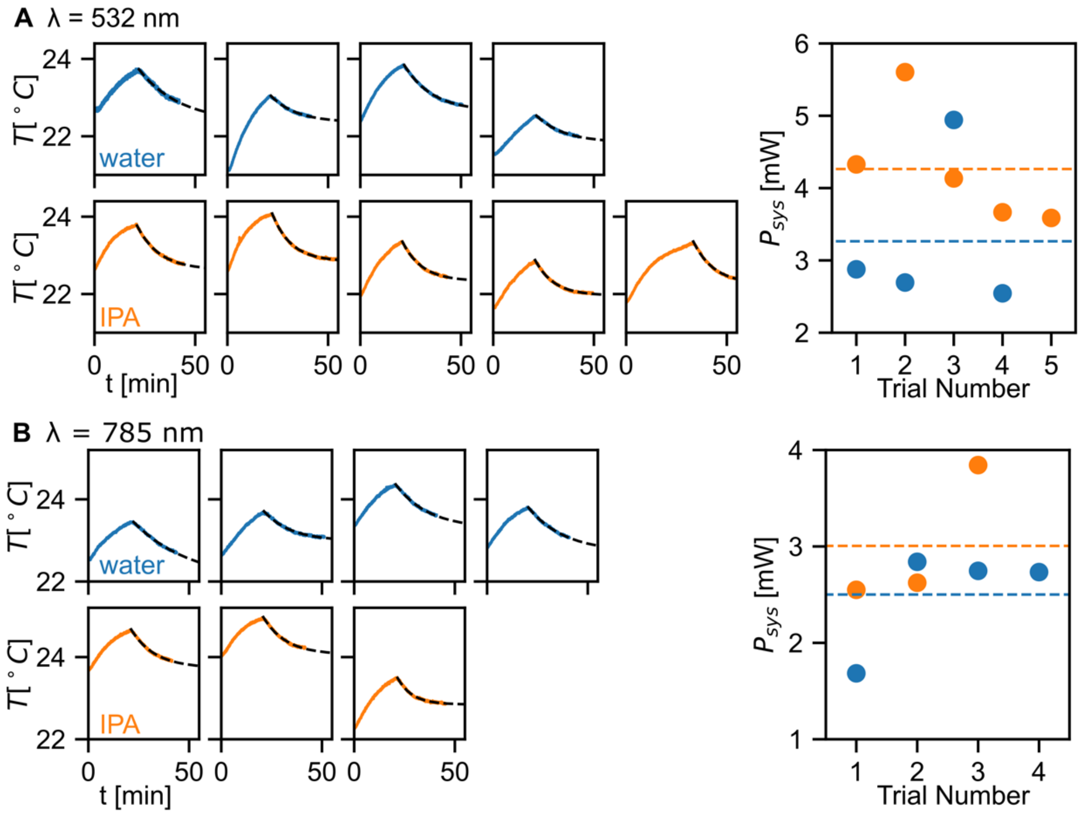


**Figure S3-1**. Measured heating and cooling curves of water (blue) and IPA (orange) upon laser illumination at 532 nm (A) and 785 nm (B) and $P_{\text{sys}}$ calculated from each trial. Differences between each trial are attributed to local changes in the environment and small differences in the optical alignment of measurement, which are proportionally larger in the $P_{\text{sys}}$ measurements compared to $P_{\text{ext}}$.

Measurements of the temperature produced by the NP suspensions and corresponding $P_{\text{ext}}$ and $\eta$ is shown in Figure S3-2 for 532 nm and Figure S3-3 for 785 nm. Each NP suspension was measured at least three times, $h_{\text{ext}}$ was extracted and $P_{\text{ext}}$ was determined. Eq. (S3-2) was used to assign an efficiency $\eta$ to each suspension.

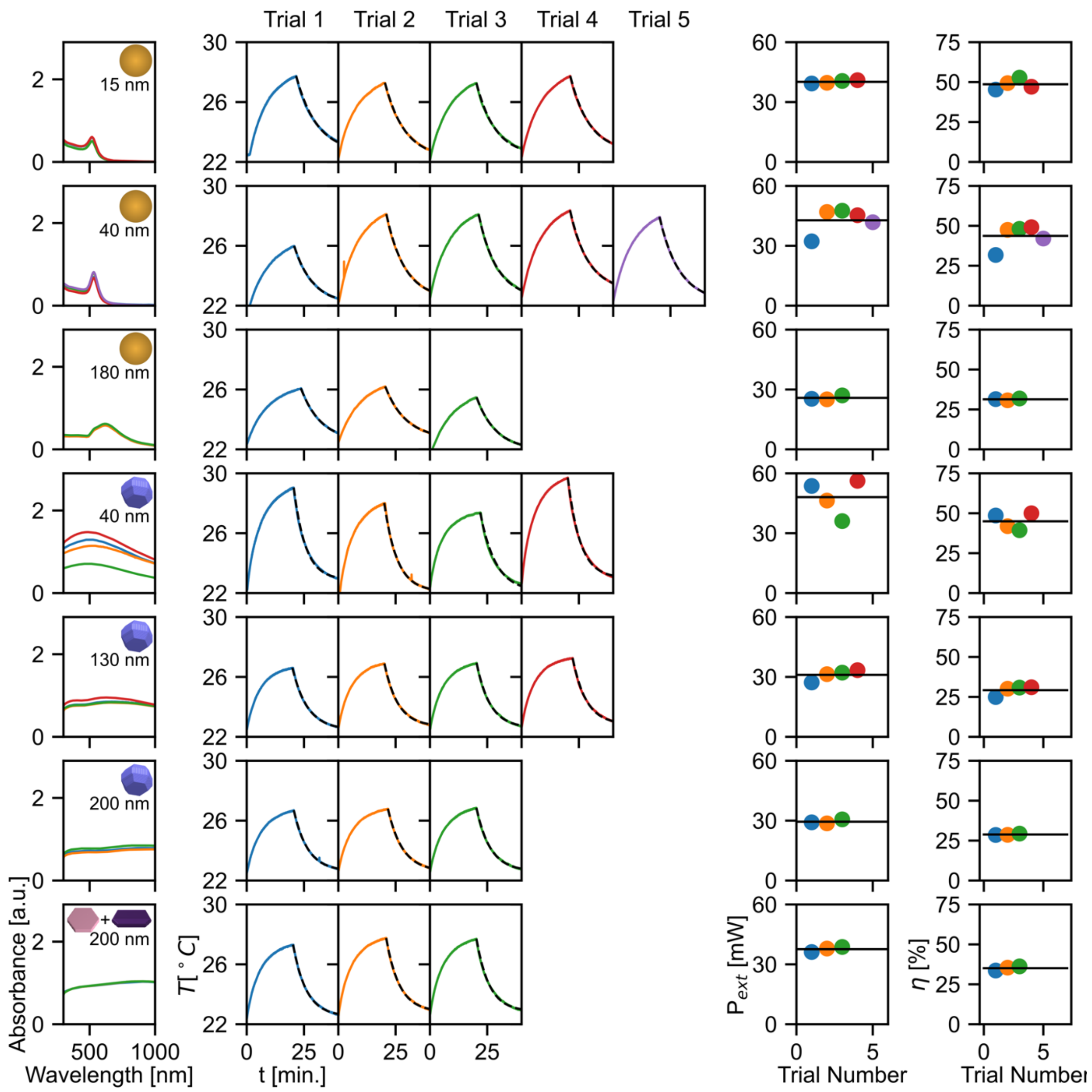


**Figure S3-2**. Measured absorbance and heating and cooling curves used to extract external heat powers and photothermal efficiencies at 532 nm excitation. The colors represent the different trials, and each row is a different category of NP suspension. The averages of $P_{\text{ext}}$ and $\eta$ are indicated by the solid line in the corresponding right panels.

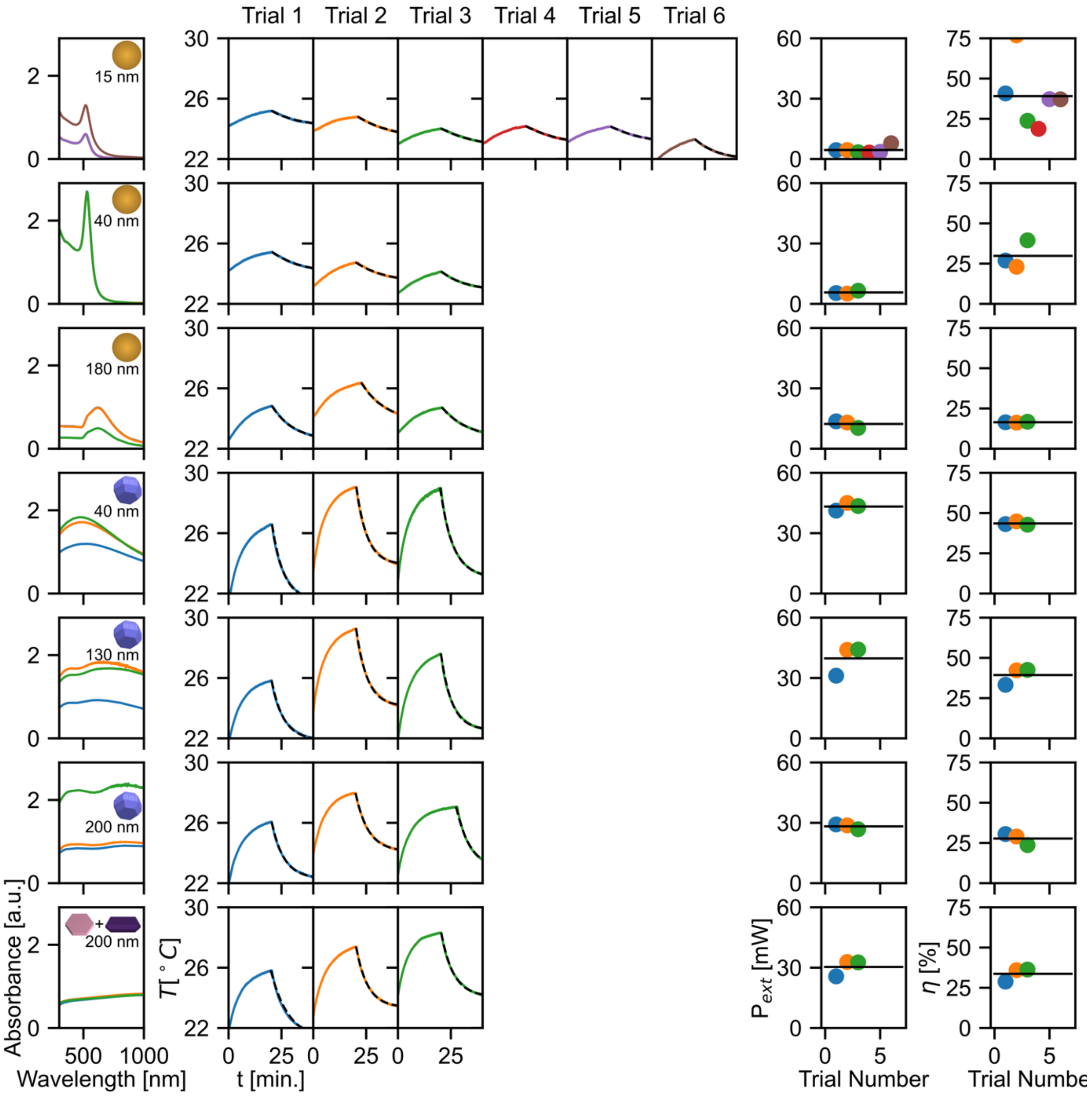


**Figure S3-3**. Measured absorbance and heating and cooling curves used to extract external heat powers and photothermal efficiencies at 785 nm excitation. The colors represent the different trials, and each row is a different category of NP suspension. The averages of $P_{\text{ext}}$ and $\eta$ are indicated by the solid line in the corresponding right panels.

## Section S4: Additional Stability Measurements of Au and Mg

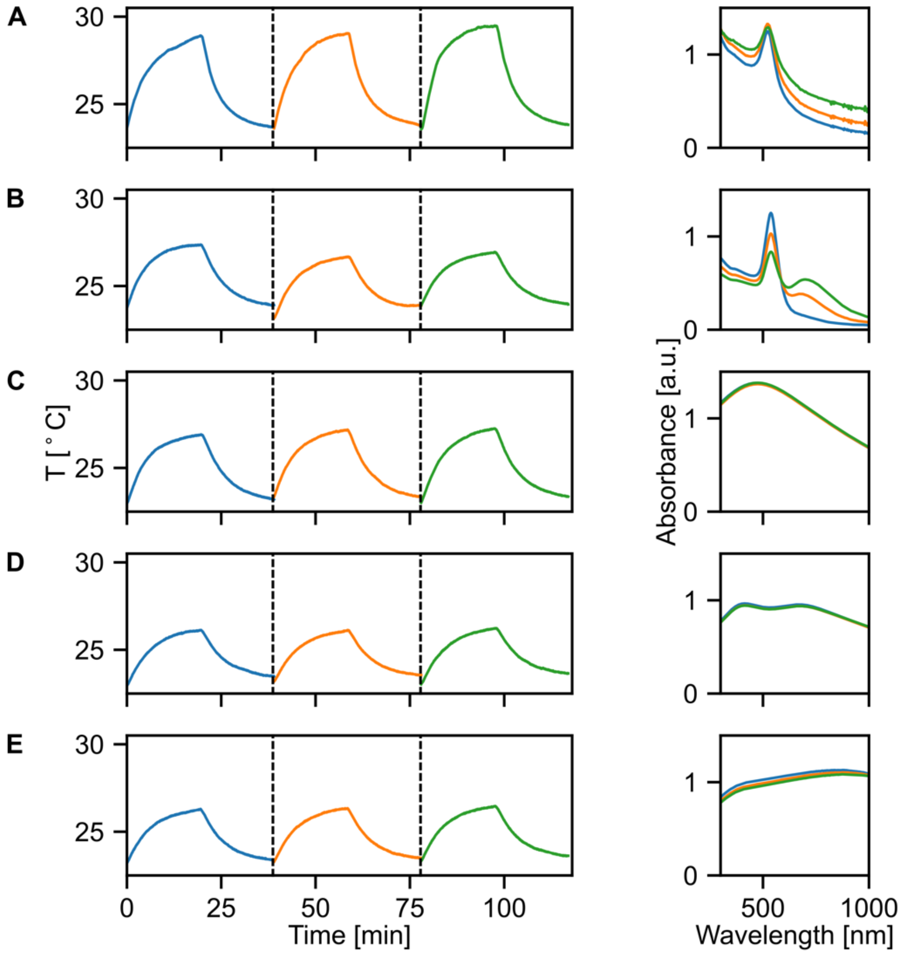


**Figure S4-1**. Stability measurements on five of the seven NP suspensions at 532 nm laser wavelength, Au nanospheres of (A) 11 nm and (B) 48 nm in diameter, Mg faceted spheroids of (C) 38 nm and (D) 200 nm diameter, and (E) Mg hexagonal platelets of 200 nm diameter. Extinction was measured after each cycle (indicated by the dashed line in temperature data), where colors in temperature panels correspond with the color traces in extinction panels.

Further stability measurements were performed on a selection of the seven NP suspensions presented in the main text: the two smallest Au NP suspensions, 11 nm (Figure S4-1 A) and 48 nm (Figure S5-1 B) diameter, the smallest and largest faceted spheroid suspensions, 38 nm (Figure S4-1 C) and 202 nm (Figure S4-1 D), and the hexagonal platelets (Figure S4-1 E). In all cases,

there was no appreciable change in the maximum temperature reached by each NP suspension after successive cycling of the heating and cooling. The changes in the Au spectra indicate minor laser-induced aggregation, which does not impact the temperature reached upon 532 nm illumination.

**References**

(1) Draine, B. T.; Flatau, P. J. Discrete-Dipole Approximation for Scattering Calculations. *J. Opt. Soc. Am. A* **1994**, *11* (4), 1491–1499.

(2) Boukouvala, C.; Ringe, E. Wulff-Based Approach to Modeling the Plasmonic Response of Single Crystal, Twinned, and Core-Shell Nanoparticles. *J. Phys. Chem. C* **2019**, *123* (41), 25501–25508. https://doi.org/10.1021/acs.jpcc.9b07584.

(3) Asselin, J.; Boukouvala, C.; Hopper, E. R.; Ramasse, Q. M.; Biggins, J. S.; Ringe, E. Tents, Chairs, Tacos, Kites, and Rods: Shapes and Plasmonic Properties of Singly Twinned Magnesium Nanoparticles. *ACS Nano* **2020**, *14* (5), 5968–5980. https://doi.org/10.1021/acsnano.0c01427.

(4) Baldwin, C. L.; Bigelow, N. W.; Masiello, D. J. Thermal Signatures of Plasmonic Fano Interferences: Toward the Achievement of Nanolocalized Temperature Manipulation. *J. Phys. Chem. Lett.* **2014**, *5* (8), 1347–1354. https://doi.org/10.1021/jz500421z.

(5) Palik, E. D. *Handbook of Optical Constants of Solid*; Academic Press, 1998.

(6) Johnson, P. B.; Christy, R. W. Optical Constants of the Noble Metals. *Phys. Rev. Lett.* **1972**, *11*, 4370–4379.

(7) Roper, D. K.; Ahn, W.; Hoepfner, M. Microscale Heat Transfer Transduced by Surface Plasmon Resonant Gold Nanoparticles. *J. Phys. Chem. C* **2007**, *111* (9), 3636–3641. https://doi.org/10.1021/jp064341w.

(8) Jiang, K.; Smith, D. A.; Pinchuk, A. Size-Dependent Photothermal Conversion Efficiencies of Plasmonically Heated Gold Nanoparticles. *J. Phys. Chem. C* **2013**, *117* (51), 27073–27080. https://doi.org/10.1021/jp409067h.

(9) Schulz, F.; Homolka, T.; Bastús, N. G.; Puntes, V.; Weller, H.; Vossmeyer, T. Little Adjustments Significantly Improve the Turkevich Synthesis of Gold Nanoparticles. *Langmuir* **2014**, *30* (35), 10779–10784. https://doi.org/10.1021/la503209b.

(10) Asselin, J.; Legros, P.; Grégoire, A.; Boudreau, D. Correlating Metal-Enhanced Fluorescence and Structural Properties in Ag@SiO2 Core-Shell Nanoparticles. *Plasmonics* **2016**, *11* (5), 1369–1376. https://doi.org/10.1007/s11468-016-0186-5.
